# Catheter-Associated Urinary Tract Infection (CAUTI)

**DOI:** 10.7759/cureus.30385

**Published:** 2022-10-17

**Authors:** Hodam Rubi, Gargi Mudey, Radha Kunjalwar

**Affiliations:** 1 Microbiology, Jawaharlal Nehru Medical College, Datta Meghe Institute of Medical Sciences, Wardha, IND

**Keywords:** catheter-associated asymptomatic bacteriuria, catheter-associated urinary tract infection, hospital acquired infection, urinary tract infections, biofilm

## Abstract

One of the most prevalent health-related illnesses globally is catheter-associated urinary tract infection (CAUTI). CAUTIs account for almost half of all hospital-acquired diseases. Most of the healthcare-acquired urinary tract infections result from catheter tubes implantation. These tubes connect a collecting system and the urinary bladder via the urethra. These are known as indwelling urinary catheters. The length of catheterization has a key role in starting bacteriuria since biofilm eventually forms on all of these devices. Despite the low percentage of people with bacteriuria who start showing symptoms, there is nevertheless a significant burden associated with these contamination due to the repeated use of indwelling urinary devices. Minimizing indwelling device usage and stopping the catheter as soon as medically possible are the two most crucial preventative measures for bacteriuria and infection when device use is required. Efforts to avoid catheter-acquired urinary infections must be implemented and monitored by infection control guidelines in healthcare institutions. These approaches include monitoring device use, the suitability of device justifications, and problems. Ultimately, technological advancements in device substances that inhibit colony generation will be necessary to avoid these infestations. There is still some way by which we can bring down the increased phenomenon of catheter-associated urinary tract contamination by maintaining hygiene while handling the catheter and patients and keeping the infected patients away or isolated from unaffected patients as a precaution. This article mainly focuses on an overview that helps with discussing prevention, risk factors, diagnosis, control and management of CAUTI.

## Introduction and background

Catheter-associated urinary tract infection (CAUTI) has been explained as the appearance of the remarkable presence of bacteria in the urine in a catheterized patient. CAUTI occurs when germs (usually bacteria) enter the urinary tract through the urinary catheter and cause infection. CAUTIs have been associated with increased morbidity, mortality, healthcare costs, and length of stay. It can be classified briefly into two: CAUTI with manifestations referable to the urinary tract and catheter-associated asymptomatic bacteriuria (CA-ASB), without expression or declaration referable to the urinary tract. One of the most typical healthcare-acquired illnesses is device-acquired urinary tract infection, accounting for up to 40% of hospital-acquired infections [1].

To prevent administering unneeded antibiotic medication, doctors must differentiate between non-manifested bacteriuria and manifested urinary tract infection; otherwise, resistance will develop against the antimicrobial drug [2]. A sizable percentage of patients receive therapy that is not advised for CAUTI after receiving an incorrect diagnosis. This ineffective therapy has the potential to be damaging due to the growth of diseases that are resistant to it, supra-infections and needless expenses. There is limited information about the manifestations and indications that identify urinary tract infection in a case with an indwelling device and how to explain or interpret CAUTI and CA-ASB. The difficulties in detecting CA-ASB have made it more difficult to reduce the incorrect treatment of the disorder. But when a CAUTI episode manifests as symptoms, there may be modest (rise in body temperature above the normal range, inflammation of the urethra, and inflammation of the urinary bladder) to serious (acute pyelonephritis, renal scarring, calculus formation, and presence of bacteria in the bloodstream) consequences. Urinary tract infections linked to catheter usage are relevant owing to their high prevalence, consequent financial expense, and potential for severe aftereffects. These infections can cause urosepsis and death if not addressed [3]. In general, indwelling urinary catheters are categorized as short-duration if they are used for fewer than 30 days and chronic or long-duration if they are used for more than 30 days.

The fundamental starting point of CAUTI is the development of a contagious or infective biofilm on the surface of the indwelling urinary catheter. The best materials for a urinary catheter are biocompatible, antibacterial, and antifouling. Patients who can be handled with intermittent catheterization versus those who need continuous indwelling catheters may have distinct demands. Given the rising antibiotic resistance in hospital-acquired infections, prevention of these infections must also be given priority [4]. They could prioritize the care of the urinary catheters if they had comprehensive knowledge of all practical preventative actions [5].

## Review

Epidemiology

Asia is expected to see an even higher CAUTI impact [6,7]. On microbiological monitoring, CAUTIs were likewise linked to increased levels of antibiotic resistance in developing nations [6,7]. The widespread use of urinary catheters in healthcare facilities emphasizes the influence and importance of CAUTI on the global and Asian healthcare systems [6-8].

In the south-eastern part of Asia, the eastern part of the Mediterranean, Europe, the western side of the Pacific, and the South and North Americas, the occurrence of catheter-acquired urinary tract contamination per 1,000 catheter days was 15.71, 9.86, 8.99, 6.90, and 5.70, respectively with a mean level of 8.50 [9]. Cases with ICU-acquired symptomatic CAUTI had a considerably greater total ICU death rate and a remarkably shorter overall ICU duration of stay than the case with ward-acquired symptomatic CAUTI [10].

The total infection rates for CAUTI, central line-associated bloodstream infection (CLABSI), and ventilator-associated pneumonia (VAP) in the ICUs of the seven hospitals that make up the International Infection Control Consortium (INICC) in seven Indian cities were 1.41 per 1,000 catheter days, 7.92 per 1,000 catheter days, and 10.46 per 1,000 ventilator days, respectively. In this study, which was conducted in ICUs of north Indian hospitals, the rate of VAP was comparatively lower while the rate of CLABSI was noticeably higher. Rates (CAUTI: 8.9/1,000 catheter days, CLABSI: 12.8/1,000 catheter days, and VAP: 24/1,000 ventilator days) were equivalent to those of 55 ICUs in underdeveloped nations [11].

Risk factor

For catheter-acquired bacteriuria, which is explained as a measurable culture with 105 organisms/ml, age, gender, physiologic score at the time of admission to hospital, period of catheterization, diabetes mellitus, immune-compromised person, neurologic disorders, and advanced systemic antibiotic exposure at the time of hospitalization are all potential risk factors [3].

Gender

 Patients with other active sites of infection and females are at much greater risk than men, and risk factors include malnutrition and renal insufficiency. Males and females both have the potential to get infected. However, male infections were more common than female infections overall. The study uncovered evidence contradicting the notion that women were more susceptible to disease because they had shorter urethra [3,12,13].

Age-Related

In geriatric hospital patients, as they already have more underlying chronic illnesses. However, it was not considered an important risk factor in the past. Until now, it is regarded as one of the risk factors causing hospital-acquired urinary tract infections, especially among those above 70 years old [13].

Period or Duration of Catheterization

The risk is further increased by placing the catheter outside the operation room after being admitted to the hospital, having a ureteral stent, and utilizing the catheter to assess urine output. Every investigation has identified extended catheterization for longer than six days as the most significant risk factor that may be modified; by the 30th day after catheterization, infection is almost ubiquitous [13,14].

In Diabetes Mellitus Patients

Due to severe neuropathy, people with diabetes mellitus have an elevated possibility of CAUTIs, which may result in insufficient bladder emptying and the colonization of microorganisms. Additionally, these patients have pancreatic beta cells that are damaged or do not generate enough insulin, which results in high sugar levels. The kidneys cannot reabsorb glucose in the presence of high glucose levels. The urine will have excessive amounts of glucose. Glucosuria affects leukocyte performance and is a medium for harmful bacteria to increase [3,12].

Other patient-related risk constituents can be poor personal hygiene, fecal incontinence, and incomplete emptying of the bladder. A urinary tract infection is quite likely in immobile inpatients. The management of urinary tract infections in the group of immobile hospitalized patients requires more focus. Continuous catheter insertion and bed rest might make a patient more susceptible to infection. There is a connection discovered between immobilization and catheter-associated tract infection. Urine flow might become stagnant if you are immobile. In an upright position, urine was moving from the renal pelvis to the bladder through the ureter due to gravity. The ureter's peristaltic cannot create gravity while the patient is lying flat. Patients may become more prone to infection if they are bedridden and have a catheter put in all the time [14]. There are factors related to caregivers like a failure in keeping up-to uncontaminated techniques both at the insertion and maintenance of catheter. Also, the catheter made up of latex has a higher risk of CAUTI [15,16].

Pathogenesis 

Causative Agents

Rectal and periurethral colonization frequently precedes catheter-associated bacteriuria, particularly in women. Thus, it is important to develop efficient preventative strategies to avoid transurethral infection [17,18].

*Escherichia coli* is the most prevalent infectious agent [1]. Additionally, often identified are additional *Enterobacteriaceae*, *Enterococci spp*, coagulase-negative *Staphylococcus*, *Pseudomonas aeruginosa*, various non-fermenters, and *Candida spp* [9].

 An organism of special significance to patients with long-term indwelling catheters is *Proteus mirabilis*. This Gram-negative rod-shaped bacterium called *Proteus mirabilis* is well recognized for producing urease, differentiating into elongated swarm cells, and moving in a unique bull's-eye pattern on agar plates. A significant source of resistant nosocomial infections is the germs that cause catheter-associated urinary incontinence [4].

Method of Invasion

Compared to gram-negative bacilli, which generated CAUTIs via both pathways equally (extraluminal, intraluminal), gram-positive *cocci* (*Enterococci* and *Staphylococci*) and yeasts were much more likely to be extraluminally acquired [17].

Urine contains large amounts of urea (400 mM), the substrate of urease, which hydrolyses it into CO_2_ and NH_3_, which is how we get rid of excess nitrogen. The released ammonia increases the urine's pH, leading to the precipitation of ordinarily soluble polyvalent anions and cations resulting in urolithiasis. Crystals can develop on and inside the catheter lumen, obstructing urine flow and requiring the removal and replacement of the catheter [18]. Patients having short-term catheterization are less likely to have this species because it is hardly isolated and secluded after the initial colonization of the catheterized urinary tract, and it seems to exist for a longer duration [19,20]. The majority of urinary tract infections linked with catheters are endogenous or caused by the patient's intestinal flora, and the catheter increases the risk of UTI in several different ways.

Drainage tubes that have been introduced via the urethra into the urine bladder are left there and then attached to a collecting system are known as indwelling urinary catheters [6]. Urinary catheter installation commonly results in infection, and daily catheter usage is correlated with an increase in bacteriuria of around 5%, which may be symptomatic or asymptomatic sometimes. Despite that, commonly it is asymptomatic, which usually does not require treatment [2]. The inner and outside planes of the indwelling devices provide pathways for bacterial invasion. The external catheter's gap with the urethral mucosa offers a path for germs to enter the bladder even when the closed system is meticulously keep-up [21]. Bacteria can get in the bladder by contaminating the catheter's tip during insertion with the distal urethra's flora or climbing along its exterior or interior. The incidence of bacteriuria is increased in catheterized individuals who have residual urine in their bladder [22,23]. Bacteria must initially cling to the urinary tract's epithelial cells and the catheter's surface to begin the infection process. Once they have developed as biofilms on the planes of the catheter, they are immune system and medication-resistant.

The bladder epithelium may suffer acute physical harm if catheters are used alone; they may also be poisonous and inflammatory. Inflammation and epithelial damage caused by bacteria may work together synergistically to induce symptoms in the patient. A biofilm is a living organism made up of several bacterial species, their produced polysaccharide matrix, and components left over from human fluids, as opposed to being a static, filmy slime layer [24,25]. Accumulation of a conditioning film on the device's superficial plane is the initial stage in the creation of a biofilm associated with catheters. The nature of the underlying surface of the catheter may be totally or partially concealed once the catheter has developed a conditioning coating. Therefore, the conditioning coating may promote microbial adhesion even if the naked catheter surface is unfavourable to colonization [24,25]. 

**Figure 1:**
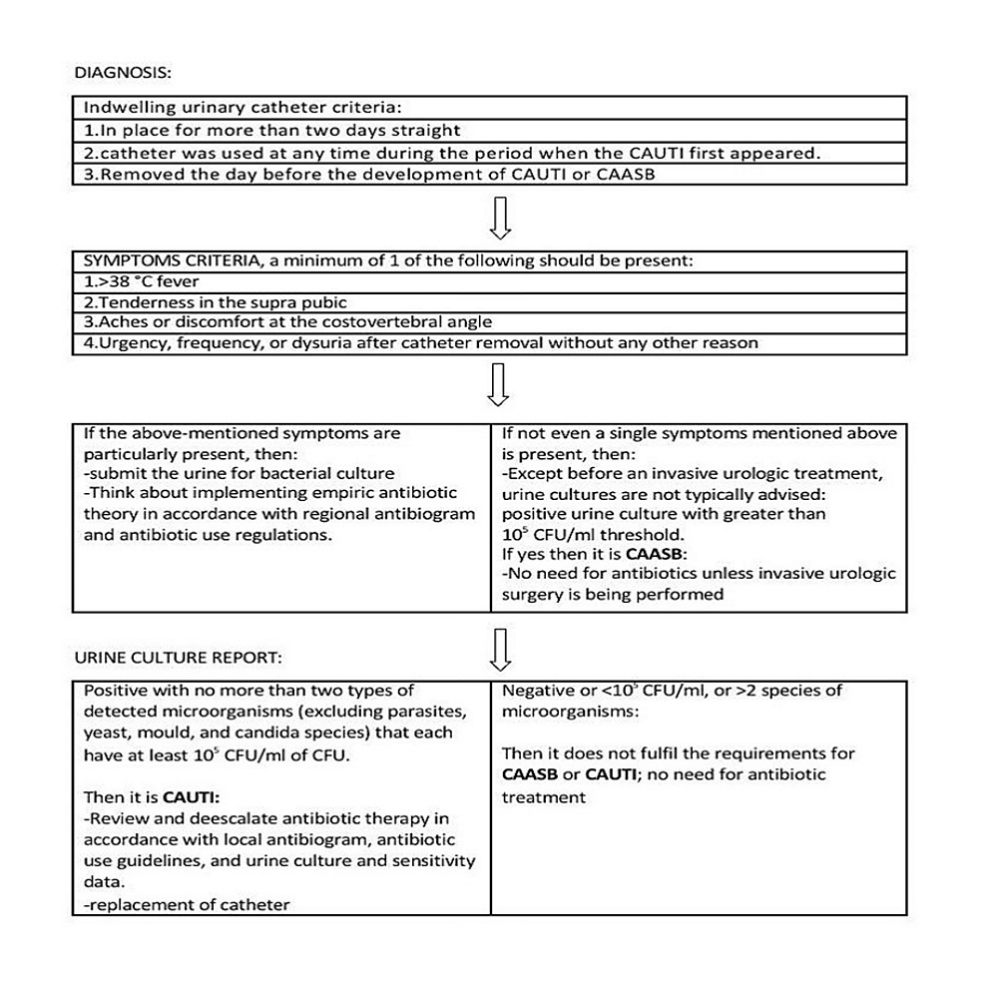
Diagnosis of catheter-associated urinary tract CAUTI: Catheter-associated urinary tract infection; CAASB: Catheter-associated asymptomatic bacteriuria Figure created by the authors.

 Figure 1 contains appropriate content that explain how to diagnose the infection that is associated with the use of catheter.

Diagnosis

Pyuria is not a sign of catheter-associated bacteriuria or CAUTI in the catheterized patient. It is not recommended to distinguish between CA-ASB and CAUTI based on the presence, absence, or intensity of pyuria. Pyuria associated with CA-ASB should not be considered a sign that an infection has to be treated with an antibiotic. When a patient presents with symptoms, the lack of pyuria points to a different diagnosis than CAUTI [26].

Prevention

Designing and assessing preventive measures requires an understanding of the etiology of CAUTI [27]. Replacing indwelling urinary catheters, other types of catheters, and other preventative methods are often categorized under these topics. Catheters with antimicrobial properties, discontinuous catheterization, suprapubic catheters, and condom catheters are potential substitutes for indwelling urinary devices that may be used to prevent CAUTI. However, the data is not yet sufficient to support these recommendations [28]. Catheterization techniques, such as condoms and suprapubic catheters, are available in addition to their selective and restricted usage. Although effective for male patients without the restriction of the bladder outlet, condom catheters need to be used carefully to prevent problems such as skin maceration and have been linked to an increased likelihood of urinary tract infections. The development of the closed catheter system, in which the drainage bag and collecting tube are joined, was essential for the decline in CAUTIs, as well as modifications to this drainage system have been made in an effort further to lower the risk of infection [29]. Choosing the right catheter size helps to reduce the chance of getting a CAUTI. To reduce CAUTIs and prevent collection bag contamination after emptying, hospital and institutional staff must properly install the catheter in an aseptic manner [14]. One of the best ways to avoid CAUTI is to ensure that the flow of urine is not impeded [27,30]. Avoiding needless catheterization days is another good way to reduce CAUTI [31]. Since biofilm on urinary devices is a key component in the pathophysiology of CAUTI, several researchers are also working on modifying the device surface to prevent the growth of biofilm [32]. The creation of silicone-based urinary catheters that are antimicrobial/antiseptic-impregnated has been the main focus of attempts to stop biofilm development [33-35].

On the other hand, the Stensballe experiment compared silicone catheters with nitrofurazone impurities to control silicone catheters and discovered that the nitrofurazone group had significantly delayed the onset of bacteriuria and experienced a reduction in the need for new or modified antimicrobial therapy [36]. Early detection and treatment of urinary tract infections have been encouraged by regular bacteriologic surveillance of catheterized patients.

A care bundle is described as the application of a collection of proof-based treatments that, when used individually, improve the healing process and outcomes for patients; when applied collectively, they produce superior results than when applied separately. Moreover, these are a set of three-to-five evidence-informed practices performed collectively and reliably to improve the quality of care. They are aimed to prevent and manage different health conditions. Preventive bundles for CAUTI are a crucial tactic in a multimodal strategy that concentrates on high-yield therapies [35,37].

Periodic in-service training should be provided to hospital staff and anyone who cares for catheters, with an emphasis on the proper practices and potential problems of urinary catheterization. Before and following manipulating the device site or equipment, hands should be washed. For insertion, you should need gloves, a drape, sponges, a suitable uncontaminated solution for periurethral cleansing, and a single-use packet of lubricating jelly. Uncontaminated and contaminated cases with indwelling devices should not share a room or beds next to each other to reduce the possibility of cross-contamination [38,39]. Approximately 35% of all healthcare-associated infections are urinary tract infections of which 80% are associated with the use of an indwelling catheter [40].

## Conclusions

The device-associated healthcare-acquired infection known as CAUTI is significant. The use of an indwelling urethral device is connected to inflated possibilities of the presence of bacteria in the blood and manifested urinary tract infections, as well as increased morbidity from non-infectious consequences. To reduce infections linked to the use of these devices, infection management programs must create, apply, and observe rules and procedures. Maintaining proper hygiene by the patient and their caretaker is also important. Choosing the right catheter also helps to reduce the chances of infection. The next best course of action is to detach the catheter shortly as it is no longer required. This action may be ordered by the computer end or finish orders that are automatically generated. In inclusion to these steps, strong adherence to infection control procedures, including making sure that urine drains according to gravity can help reduce CAUTI. All of the aforementioned actions may be taken right away and cost minimal money. The attempts to restrict the application of urinary catheters are admirable and reasonable. As a result, in the years to come, the issue of by what method to control the catheter-related presence of bacteria in urine and CAUTI will only grow in importance. For the present and upcoming generations of medical researchers, the unresolved issues in this field should serve as an inspirational challenge.
